# Mechanical, Durability, and Microstructure Assessment of Wastepaper Fiber-Reinforced Concrete Containing Metakaolin

**DOI:** 10.3390/ma17112608

**Published:** 2024-05-28

**Authors:** Mohammad Valizadeh Kiamahalleh, Aliakbar Gholampour, Mohsen Rezaei Shahmirzadi, Tuan D. Ngo, Togay Ozbakkaloglu

**Affiliations:** 1 College of Science and Engineering, Flinders University, Tonsley, Adelaide, SA 5042, Australia; mohammad.valizadeh@flinders.edu.au; 2 Department of Infrastructure Engineering, University of Melbourne, Parkville, Melbourne, VIC 3052, Australia; mohsen.rezaeishahmirzadi@unimelb.edu.au (M.R.S.); dtngo@unimelb.edu.au (T.D.N.); 3 Ingram School of Engineering, Texas State University, San Marcos, TX 78666, USA; togay.oz@txstate.edu

**Keywords:** plasterboard, wastepaper fiber, metakaolin, concrete, crack propagation, microstructure

## Abstract

This study evaluates the potential use of discarded plasterboard paper as fibers from buildings to reinforce concrete. Various concentrations of wastepaper fibers (0.5%, 1%, 1.5%, 2%, and 2.5% by weight of the binder) were investigated in this research. To mitigate the water absorption effect of the paper fibers, metakaolin was employed as a partial cement replacement. The results demonstrate that the inclusion of the wastepaper fiber enhances the mechanical and durability performance of the concrete. The optimal fiber proportion was identified as 1%, leading to a 29% increase in the compressive strength, a 38% increase in the splitting tensile strength, a 12% decrease in the water absorption, and a 23% decrease in the drying shrinkage with respect to the concrete containing 20% metakaolin. However, exceeding this optimal fiber content results in decreased mechanical and durability properties due to the fiber agglomeration and non-uniform fiber distribution within the concrete matrix. Based on the microstructural analysis, the improved performance of the concrete is ascribed to decreased porosity, more refined pore structure, and reduced propagation of microcracks within the concrete matrix in the presence of wastepaper fiber. According to the results, concrete containing 20% metakaolin and 1% wastepaper fiber exhibits durability and mechanical properties comparable to those of the traditional concrete. This finding highlights the significant promise of reducing dependency on conventional cement and incorporating suitable recycled materials, such as discarded plasterboard, and secondary by-products like metakaolin. Such a strategy encourages the preservation of resources, reduction in carbon dioxide emissions, and a decrease in the ecological footprint resulting from concrete production.

## 1. Introduction

At present, a significant amount of concrete is being employed in the building industry because of its benefits, such as ease of shaping, great compressive strength, and long-lasting qualities. Nevertheless, the continuous use of cement in concrete manufacturing is leading to an increase in carbon emissions [1]. Consequently, the field of concrete technology faces ongoing challenges to develop innovative concrete formulations to reduce these emissions [2]. The annual production of the construction and demolition (C&D) waste globally exceeds 10 billion tons. Managing this waste and mitigating its environmental impact have become significant and pressing challenges worldwide [3]. The primary constituents of the C&D waste consist of concrete, asphalt, plasterboard, wood products, and masonry. Additionally, it often contains notable amounts of plastics, metals, earth, insulation, shingles, additives (e.g., dyes), as well as paper and cardboard [4,5].

Plasterboard as one of the major C&D waste is made by enclosing gypsum within two layers of laminated cellulosic paperboard [6]. Annually, a worldwide output of 80 million tons of plasterboard is generated, with a substantial portion of it being disposed of in landfills as a C&D waste [7]. Composed of gypsum powder, paper, and a minor amount of additives, plasterboard waste is fully capable of being recycled. Recycling plasterboard not only diverts valuable waste from ending up in landfills but also reduces the costs associated with landfill management and waste transportation. Moreover, it aids in the conservation of natural resources by diminishing the demand for gypsum, a naturally occurring mineral with limited availability [8]. Reprocessing the waste plasterboard can generate high-quality gypsum, meeting specific standards and offering diverse applications such as cement manufacturing, road sub-base construction, and agricultural soil improvement, distinct from its use in creating new plasterboard [9].

During the recycling process of the plasterboards, it is necessary to separate the gypsum core from the two layers of paper [10]. However, the paper that serves as a covering for plasterboard is presently ignored and discarded in landfills, where it ultimately undergoes incineration. One potential approach to recycle this wastepaper obtained from discarded plasterboard involves its use as a reinforcing fiber material within concrete. The recycling of the wastepaper as a fiber within the concrete offers an effective solution to both the building sector and environmental preservation [11]. Converting one metric ton of the wastepaper is equivalent to safeguarding 17 trees, conserving 4102 kilowatt-hours of energy, preserving 2600 L of water, and averting the emission of 27 kg of hazardous substances into the atmosphere [12].

The existing literature contains only a few studies on the recycling of paper fibers in cementitious materials (e.g., paste [13], mortar [14,15], and concrete [16,17,18,19]). Previous studies have employed paper fibers sourced from discarded office paper, newspapers, packaging materials, cardboard, magazines, and pamphlets in the cementitious materials [11]. It is important to highlight that paper fibers, being of a cellulosic nature, are appreciated for their strength, flexibility, and their ability to create a cohesive network when combined with cementitious materials. Cellulosic fibers are recognized for their capacity to reduce thermal conductivity, enhance acoustic properties through increased sound absorption, decrease density, increase flexural strength, and control the initiation and growth of microcracks of concrete [20]. Zaki et al. [21] conducted a slump test to assess fresh properties of concrete that incorporated varying amounts of wastepaper from institutional paper sources (e.g., schools, administration offices, and libraries) as a partial substitute based on the weight of cement. It was observed that the slump increased when 5% wastepaper was used. Chung et al. [22] evaluated lightweight concrete with waste newspaper as an aggregate and found that higher wastepaper-to-cement (P/C) ratios resulted in a lower density, compressive strength, and elastic modulus and a higher ductility in the concrete. Jung et al. [23] substituted cement with wastepaper in mortar ranging from 0% to 20% replacement relative to the cement weight, and found that 5% replacement developed the highest compressive strength due to the largest amount of hydration products in the mix. Ilakkiya and Dhanalakshmi [16] conducted an assessment of the splitting tensile strength in concrete mixes with commercial wastepaper sourced from writing, printing, packaging, as well as industrial and construction applications. Their findings indicated an improvement in the tensile strength for the concrete with 5% and 10% wastepaper, relative to the unmodified control mix. Praburanganathan et al. [2] explored the impact of partial cement replacement with discarded wastepaper (ranging from 0% to 20%) on the durability and mechanical characteristics of concrete. They revealed that increasing wastepaper content led to an increase in the water absorption of concrete, and 5% wastepaper developed the highest compressive strength.

Although wastepaper improves mechanical properties of cementitious materials, the high-water absorption of the wastepaper limits its application. Cellulosic fibers within cementitious materials experience degradation when subjected to humid conditions, leading to a decrease in their mechanical properties over time [24]. This deterioration is caused by alkaline decomposition and mineralization of the reinforcing fibers, resulting in a decrease in the overall tensile strength of the cementitious materials and a decrease in the adhesion between the fibers and the cementitious matrix after cracking [25]. Numerous research investigations have employed metakaolin as either an additive or a partial replacement for cement to enhance durability and mechanical characteristics of concrete [26,27]. Owing to its substantial pozzolanic influence, the incorporation of metakaolin into concrete causes a significant enhancement in the compressive strength of the cementitious materials over a long period [28]. When metakaolin is added to concrete mixes, it reacts with the calcium hydroxide released during cement hydration, forming supplementary calcium silicate hydrate gel [27,29]. This reduces the overall water absorption of the material [30], which in turn limits the volume changes and dimensional instability typically associated with cellulosic fibers, thus mitigating degradation due to excessive moisture. Therefore, incorporating metakaolin into the wastepaper in concrete can enhance the durability problems associated with the wastepaper [31]. Based on the above literature review, it is evident that while the inclusion of wastepaper fibers in cementitious materials offers mechanical and sustainability benefits, there remains a gap in understanding how these fibers interact with pozzolanic materials such as metakaolin in concrete applications. The potential of metakaolin to improve mechanical properties and address the durability issues presented by the high-water absorption of wastepaper has not been fully explored. This research aims to fill this gap by evaluating the impact of wastepaper fibers from waste plasterboard and metakaolin on the mechanical strength and durability of concrete, providing a novel insight into sustainable building materials.

In response to the above-mentioned research gap, metakaolin was used as a partial cement replacement in this study to mitigate the high-water absorption effect of the wastepaper on the properties of concrete. Determining the wastepaper fiber proportion that equates to or surpasses the performance of conventional concrete in terms of mechanical strength and durability was the primary focus of this study. A series of experiments were conducted, including compressive and tensile strengths, as well as water absorption and drying shrinkage tests. Microstructural analysis was also performed on the concretes using scanning electron microscopy (SEM) and energy dispersive X-ray spectroscopy (EDS) after a curing period of 28 days. This study presents a novel approach, utilizing metakaolin as a substitute for cement in wastepaper fiber-reinforced concrete. This marks the first investigation in the literature on using wastepaper fiber derived from waste plasterboard, aiming to determine the optimal ratios for developing concrete with mechanical and durability properties comparable to conventional concrete.

## 2. Materials and Methods

**Figure 1:**
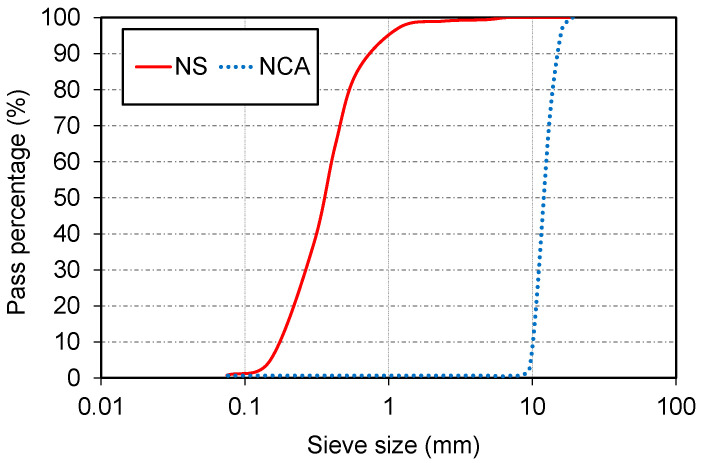
Sieving analysis of fine aggregates: NS: natural sand, NCA: natural coarse aggregate.

Cement and metakaolin were used as binder materials. The cement, an ordinary Portland cement, exhibited a dark grey hue, and had a specific gravity of 3.176. In contrast, the metakaolin was plain white in color, and demonstrated high fineness with passing percentages of 99, 98, and 97% for particle sizes of 300, 150, and 75 µm, respectively. The specific gravity of the metakaolin was 2.670. The cement was supplied from Adbri, Sydney, Australia, which was produced based on AS3972 [32] for general purpose. Metakaolin was a commercial material supplied from Boral, North Ryde, Australia. The chemical composition of the cement and metakaolin is presented in Table 1. The river sand had a creamy-yellow color. It had an upper particle size limit of 2.36 mm, a bulk density of 2610 kg/m^3^, and a water absorption rate of 2.24%. The coarse aggregate was bluestone and had a maximum size of 16 mm. Its bulk density was recorded at 2720 kg/m^3^, and it had a water absorption of 0.35%. Figure 1 presents the sieving analysis of the aggregates. Superplasticizer MasterGlenium SKY 8700 was used to improve the workability of mixes. The wastepaper fiber was derived from discarded plasterboard provided by Sunshine Groupe, Victoria, Australia. It had a density of 50 kg/m^3^ and a water absorption rate of 824%.

Table 2 provides proportions of different concrete mixes. A total of seven concrete mixes were prepared, including one control mix with 100% cement and six mixes in which 20% of cement was replaced with metakaolin. Nezerka et al. [33] reported that using more than 20% metakaolin significantly decreased the strength of concrete mixes. Therefore, it was decided to use 20% metakaolin in the mixes. Additionally, the sand was used in a saturated surface dry (SSD) state to mitigate its water absorption in the concrete mixes. The preparation of the mixes involved thorough blending of binder, sand, and aggregate for 2 min. Following this, the wastepaper fiber was gradually introduced and mixed for an additional 2 min to ensure uniform distribution. Subsequently, water and superplasticizer were slowly added at 50%, 75%, and 100% increments and mixed for 3 min. The resulting materials were then poured into the appropriate molds, and the prepared specimens were demolded after 24 h.

In the six metakaolin mixes, wastepaper fibers were incorporated at binder weights of 0, 0.5, 1, 1.5, 2, and 2.5%. The control mix was designed to attain the compressive strength of 35 MPa at 28 days of curing. The water-to-binder (w/b) ratio of 0.60 was considered for all mixes. 

To distinguish the mix identification in Table 2, control stands for the control mix, MP is the label for mixes containing metakaolin, and the numbers associated with MP indicate the percentage of the plasterboard wastepaper fiber included in each mix. For example, MP2.0 denotes a concrete mix with 80% cement and 20% metakaolin as binder and 2% wastepaper fiber as reinforcement.

**Figure 2:**
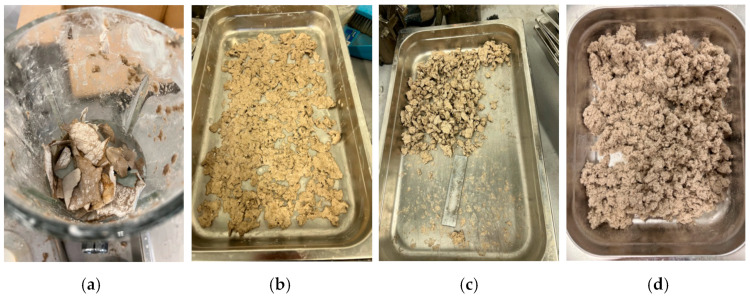
Production of wastepaper fiber from waste plasterboard. (**a**) Blending wastepaper from plasterboard; (**b**) Dry out pulp to remove moisture; (**c**) Collect and blend dry material without water; (**d**) Final product.

**Figure 3:**
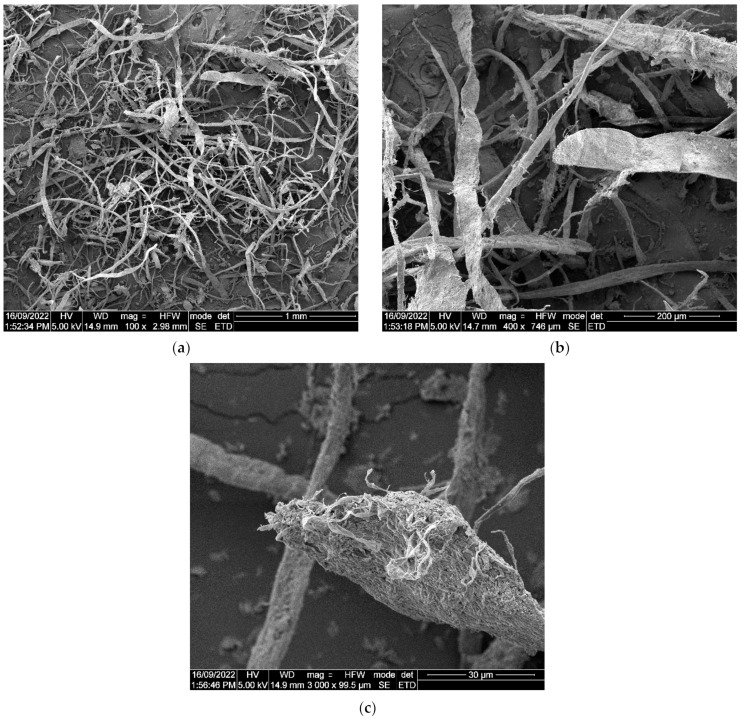
SEM images of prepared wastepaper fibers at different magnifications. (**a**) 100×; (**b**) 400×; (**c**) 3000×.

The process for preparing waste plasterboard fiber from discarded paper is illustrated in Figure 2, and it aligns with the procedure described in the literature [34]. As shown in Figure 2, the initial step involved immersing the waste plasterboard in a container filled with an appropriate amount of water. Subsequently, the soaked plasterboard was broken down into smaller fragments using a blender for 15 s. After blending, any excess water was removed from the finer materials. Next, the resulting moist paper fibers were subjected to a 24 h drying cycle in an oven, forming a cohesive block. This dried paper fiber was then reintroduced into the blender for an additional 30 s to further reduce its size. The resulting wastepaper fiber was characterized by lengths ranging from 1 to 4 mm, an average diameter of 70 µm, and an aspect ratio ranging from 5 to 14. Figure 3 also displays the SEM images of the prepared wastepaper fibers at various levels of magnification.

**Figure 4:**
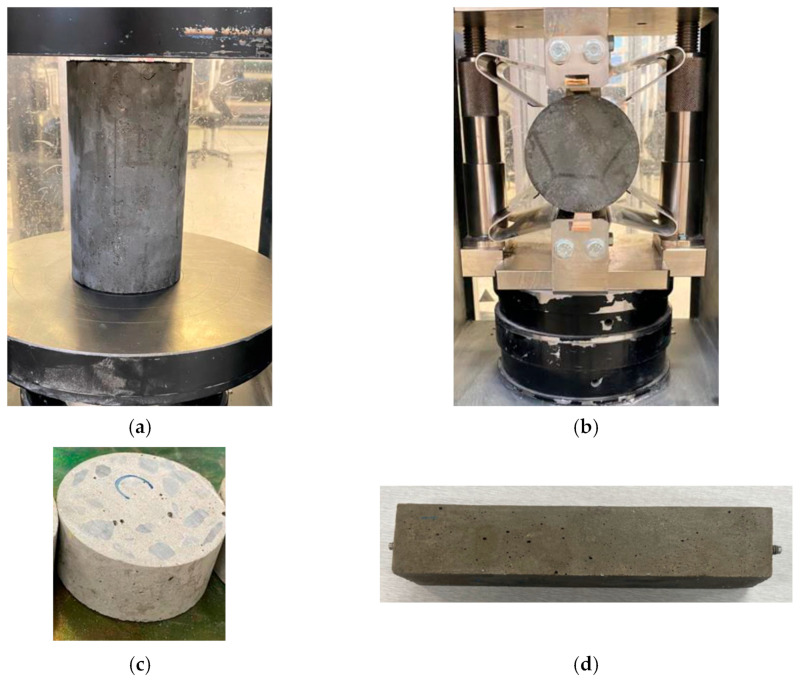
Photos of (**a**) axial compression, (**b**) splitting tension, (**c**) water absorption, and (**d**) drying shrinkage test samples.

The slump test on fresh concrete mixes was done in agreement with ASTM C143/C143M [35]. Axial compressive strength testing, following ASTM C39 [36], and splitting tensile strength assessment, in agreement with ASTM C496 [37], were carried out on hardened cylindrical specimens measuring 150 × 300 mm. The water absorption test was performed on cylindrical specimens measuring 150 × 50 mm, following ASTM C642 [38]. Drying shrinkage testing was carried out on prism-shaped specimens measuring 40 × 40 × 160 mm, as per ASTM C157/C157M [39]. Each mix and property were tested using three nominally similar specimens. The strength and water absorption tests were done at 28 days of curing and the drying shrinkage test was done at 0, 4, 8, 12, 16, 20, 24, and 28 days of curing. Figure 4 displays images from various tests. The SEM and EDS examinations were carried out utilizing the FEI Inspect F50 SEM (Hillsboro, OR, USA). Three replicate samples were prepared for each test, and the average of these three tests was calculated to determine the final result.

## 3. Results

### 3.1. Slump

**Figure 5:**
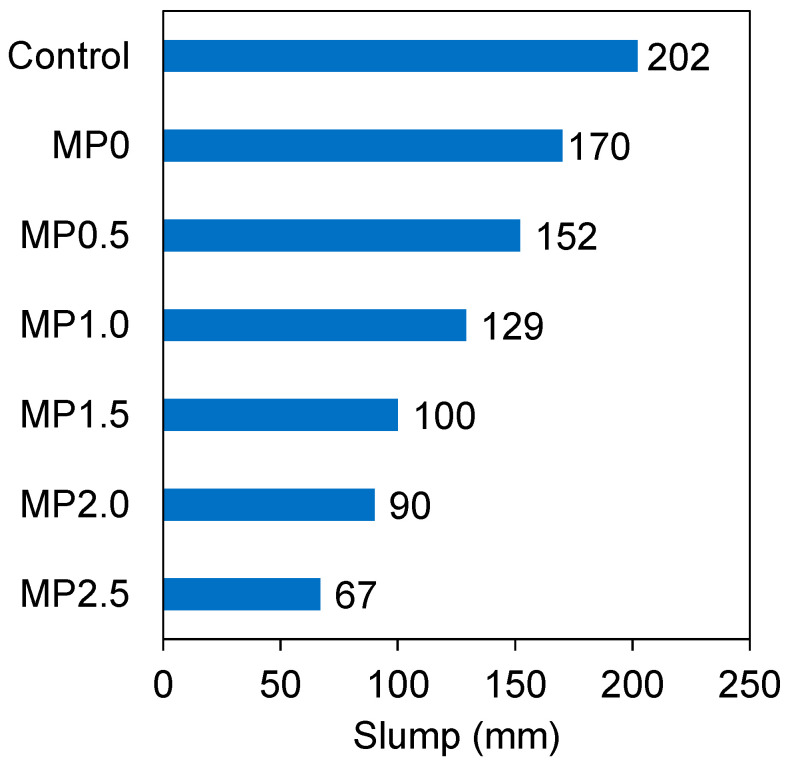
Workability of different mixes.

Figure 5 illustrates the slump measurements for different mixes. It is evident that the conventional concrete had the greatest slump value. However, when 20% metakaolin was integrated into the mix, there was a slump decrease of approximately 16%. This observation agrees with previous studies [40,41]. The irregular shape and substantial specific surface area of the metakaolin particles leading to the increased friction forces among the cementitious particles contribute to the decreased workability [42]. Barkat et al. [43] conducted a study to examine how the inclusion of metakaolin affects the performance of self-compacting limestone cement concrete. Their research revealed that when 15% metakaolin was used as a replacement for cement, the workability decreased by approximately 38%. They attributed this decrease in the workability to the increased surface area of the binder resulting from the metakaolin incorporation.

As depicted in Figure 5, the addition of wastepaper fiber resulted in a decrease in the slump. The MP0.5, MP1.0, MP1.5, MP2.0, and MP2.5 mixes displayed slump values 11, 24, 41, 47, and 61% lower than the MP0 mix, respectively. This finding aligns with prior studies [44,45]. This correlation arises from the fact that cellulose, the primary constituent of the wastepaper, is a hydrophilic material known for its exceptional water-absorbing properties [46]. This factor, in combination with the irregular geometry and rough texture of the fiber, has the potential to hinder the mobility of concrete constituents during mixing, resulting in an additional reduction in workability [47]. Balwaik et al. [48] investigated the use of commercial wastepaper pulp, sourced from paper mills, as a partial replacement for cement in concrete. They observed a 39% decrease in the workability when 20% of the cement was replaced with the pulp. They ascribed this outcome to the physical characteristics of the paper pulp.

### 3.2. Compressive Strength

**Figure 6:**
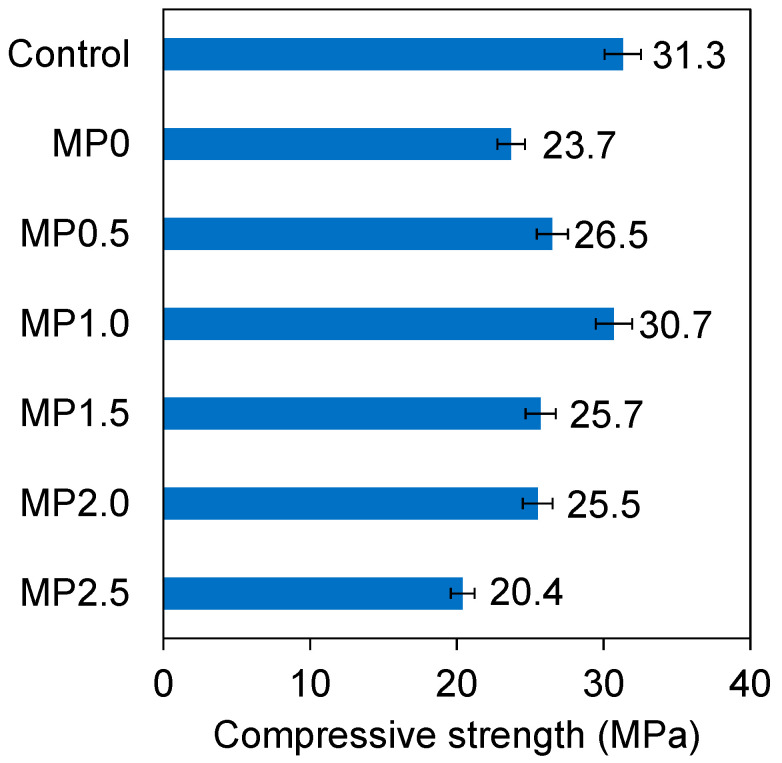
Compressive strengths of different mixes. The error bars represent deviation from the mean values.

Figure 6 illustrates the mean compressive strength of the mixes following a 28-day curing. The inclusion of 20% metakaolin resulted in a 39% decrease in the compressive strength of the mix. As will be discussed later under microstructure analysis, the substitution of cement with metakaolin in the concrete mix had a detrimental impact on the structural stability of the concrete. The incorporation of 20% metakaolin led to an increase in pore count and an increased prevalence of microcracks in contrast to the control mix with a more compact structure. This trend is consistent with the previous research finding on the compressive strength development of high performance concrete containing 10% of metakaolin as cement replacement, even though this study indicated that the long-term effects of metakaolin inclusion could be beneficial due to secondary pozzolanic reactions [49]. The reduced strength can be attributed to various factors, including reduced cement hydration affecting the binding material, delayed nucleation of calcium silicate hydrate within the mix [50], and the dilution effect caused by metakaolin, which functions as a pozzolanic material and introduces additional inert particles into the mix [51]. The metakaolin particles are exceptionally tiny, characterized by a high surface energy, causing them to agglomerate. These agglomerations consist of a random assortment of particles, with low compactness and trapped air. As a result, these clusters act like larger particles with decreased reactivity. Hence, the decrease in the compressive strength when the metakaolin content increases can be related to the presence of these agglomerated structures [52].

As depicted in Figure 6, the introduction of the wastepaper fibers resulted in a notable improvement in the compressive strength of the mixes. Specifically, a 1% increase in the wastepaper fiber content was correlated with a 29% increase in compressive strength for the metakaolin mix, making the strength of the MP1.0 mix comparable to that of the control mix. As will be indicated by the SEM, this improved strength is attributable to the wastepaper fibers significantly reducing pore numbers and microcracks, which in turn resulted in a denser concrete mix. Furthermore, the wastepaper fibers were distributed consistently throughout the concrete matrix, which played a key role in minimizing the size and spread of microcracks (facilitated bridging effect), thus fostering a uniform distribution of stress within the matrix.

Nevertheless, when the wastepaper fiber content exceeded 1%, a decrease in the strength was observed. The decrease in the strength observed with a greater fiber content beyond the optimal threshold is a consequence of fiber clustering and irregular dispersion within the concrete matrix. This phenomenon contributes to the formation of voids and cracks in the concrete mix [53]. The research of Praburanganathan et al. [2] involved an examination of the use of sun-dried preprocessed discarded wastepaper as a replacement for cement in concrete production, ranging from 0% to 20% by weight. The study unveiled that using 5% of the paper contributed to improved compressive strength by about 30%, while exceeding this threshold led to a decrease in the strength of the concrete. In their study, Lee et al. [46] explored the influence of incorporating waste newspaper as a cement substitute, ranging from 0% to 1.0%, on a mortar. Their research indicated that the 28-day compressive strength of the mortar with waste newspaper increased approximately 3–16% when compared to the control mortar.

### 3.3. Splitting Tensile Strength

**Figure 7:**
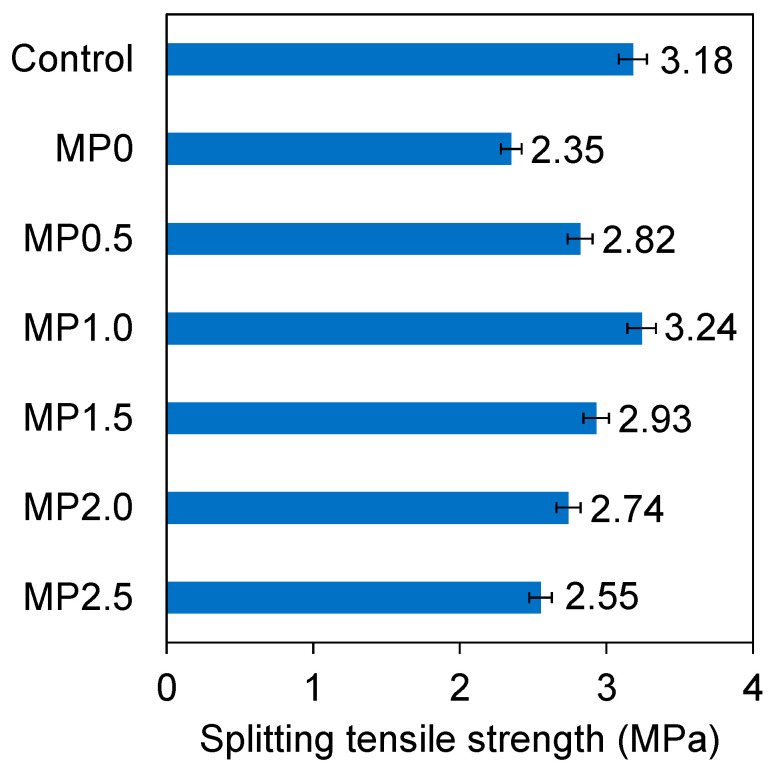
Splitting tensile strengths of different mixes.

In Figure 7, the results of the splitting tensile strength of various mixes at 28 days of curing are presented. As illustrated, there was a 26% decrease in the splitting tensile strength of the concrete when 20% of the cement was substituted with metakaolin. This finding is consistent with a prior investigation of high-performance concrete [54]. Despite the well-known pozzolanic activity of metakaolin, which can enhance concrete strength, it necessitates a certain duration for the pozzolanic reaction to take place. Consequently, its contribution may not be as immediate as that of cement [55]. The slower reaction rate of the metakaolin-containing mixes, in contrast to conventional concrete, may be attributed to the low concentration of Ca(OH)_2_ available for the pozzolan to react with [55]. Moreover, the particle size and shape of metakaolin could potentially impact the compactness and bonding within the concrete matrix, thereby influencing the tensile strength of the concrete [56].

Previous research indicated that exceeding 15% metakaolin content could increase porosity within the concrete mix [57]. However, the study carried out by Khatib and Clay [57] provides insight into the complex behavior of concrete with high levels of metakaolin substitution. Specifically, it was found that while water absorption capacity, defined as the water absorption coefficient, decreased with increasing percentages of metakaolin up to 14 days of curing, a slight increase in the water absorption coefficient was observed between 14 and 28 days, especially at a 20% replacement level. Despite this, the water absorption coefficient remained lower than that of the control mix even at this higher substitution rate, and no water was observed on the top surface of samples containing 15% and 20% metakaolin after capillary water absorption tests, suggesting a pore-blocking effect that could be beneficial in certain applications [57,58]. This temporary increase in the water absorption coefficient was attributed to changes in phase composition during the curing process, potentially leading to a denser hydration phase and could explain the variation in water absorption observed with time. At 90 days, concrete with 10% to 20% metakaolin still demonstrated a decrease in the water absorption coefficient compared to the control mix. A report by Larbi and Bijen [59] indicated a decrease of over 50% at 100 days for concrete containing 20% metakaolin. Such findings provide a partial justification for the use of a 20% metakaolin replacement level in this study, aiming to explore its long-term effects on concrete properties, including tensile strength and durability, despite the initial decrease in the strength observed in the tests. Zhang et al. [60] observed that metakaolin contents exceeding 10% could intensify the clinker dilution effect, leading to the encapsulation of cement particles, hindering the beneficial cement hydration process and decreasing the occurrence of calcium silicate hydrate in the matrix. The present study sought to investigate the threshold at which these effects become critically detrimental to the tensile strength of the concrete. By choosing a 20% replacement level, this aimed to extend the understanding of the influence of metakaolin on the concrete microstructure, especially since the literature indicates conflicting outcomes for this replacement level. Gadag et al. [54] remarked that increasing the replacement of cement with more than 10% metakaolin in high-performance concrete resulted in a decrease in the splitting tensile strength. Their findings indicated that the primary factor contributing to the degradation of the microstructure was the significant presence of unreacted metakaolin particles at increased replacement levels. The present study aligns with these findings at the 20% level, and further analyzes the trade-off between the pozzolanic benefits and the potential for increased porosity and microstructural degradation at this higher concentration of metakaolin.

Figure 7 provides evidence that the incorporation of wastepaper fiber into metakaolin-containing concrete significantly improved its splitting tensile strength. Adding 0.5 and 1% wastepaper fiber increased the splitting tensile strength by approximately 20 and 38%, respectively, compared to the MP0 mix. Moreover, the concrete containing 1% wastepaper fiber attained a splitting tensile strength equivalent to that of the control sample. According to the SEM images presented later, the inclusion of the fibers in the concrete matrix effectively bridged the microcracks and inhibited their progression, ultimately enhancing the splitting tensile strength. Moreover, even distribution of the fibers consistently helped to reduce the dimensions and extent of microcracks and quantity of pores, promoting a uniform stress distribution within the concrete structure. The tensile properties of cellulose fiber-containing concretes can be influenced by the effectiveness of load transfer between the fibers and the cementitious matrix [61].

Nevertheless, as can be seen in Figure 7, any additional increase in the wastepaper fiber content beyond 1% resulted in a decrease in the splitting tensile strength. The inclusion of 1.5, 2, and 2.5% wastepaper fiber led to a decrease of 9, 15, and 21% in the splitting tensile strength, respectively, in comparison to the 1% wastepaper fiber content. The splitting tensile strength attained with 2.5% wastepaper still exceeded that of the MP0 mix, which lacked wastepaper. These findings indicate that while the initial introduction of the wastepaper fiber enhanced the splitting tensile strength of the metakaolin concrete, potentially attributing to improved crack resistance, there exists an optimal threshold beyond which additional wastepaper fiber incorporation may have adverse effects. This effect could be attributed to an oversaturation of the concrete matrix with fibers, as will be demonstrated later by the SEM images, resulting in the fiber agglomeration. The fiber agglomeration resulting in the formation of the fiber balls hinders the effective fiber dispersion and compromises the mechanical strength of concrete by introducing weak points within the matrix [61]. Furthermore, the increased wastepaper fiber levels could lead to an increased water demand, and, consequently, a decrease in the workability of the mix, as was evidenced by the slump test results. The obtained results align with the previous research on the utilization of wastepaper in concrete [62]. Seyyedalipour et al. [63] conducted an assessment on the incorporation of waste materials from the pulp and paper industry in concrete production. Their study revealed that adding 5% wastepaper (by weight of cement) resulted in a 7% improvement in the splitting tensile strength of the concrete, but additional increments in the wastepaper content led to a decrease in the splitting tensile strength. Lee et al. [46] conducted a study to investigate the impact of substituting cement with waste newspaper in mortars. Their findings demonstrated that the 28-day splitting tensile strength of the mortar incorporating 1% waste newspaper exhibited an increase of approximately 8% when compared to that of the control mortar. In a study conducted by Alomayri et al. [64], it was discovered that incorporating 0.3% (by volume of concrete) jute and banana fiber into high-performance concrete led to an increase of around 20% in the splitting tensile strength when compared to the control concrete.

To evaluate the suitability of current design codes in estimating the splitting tensile strength of concrete mixes incorporating wastepaper fiber, this study involved a comparison of the experimental results with predictions from the codes. The design code expressions for estimating the splitting tensile strength of conventional concrete are detailed in Table 3. In the table *f’_c_* is the compressive strength of concrete.

**Figure 8:**
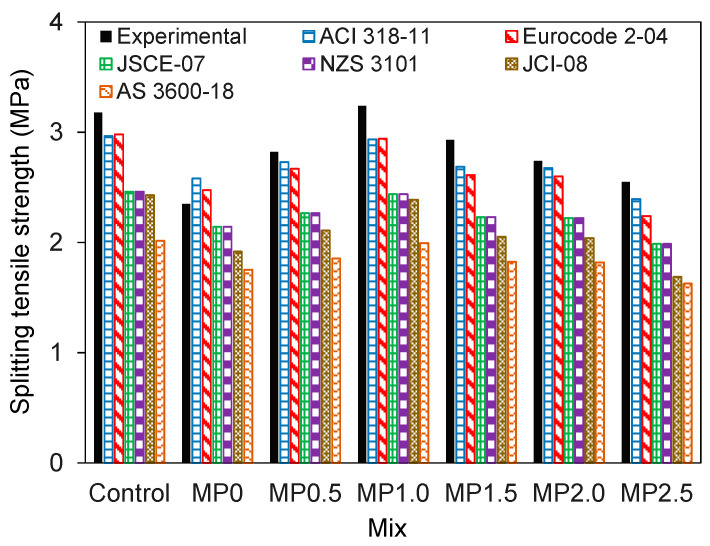
Comparison of experimental values and design code predictions for splitting tensile strength of different mixes.

Figure 8 illustrates a contrast in the splitting tensile strength among the mixes from experimental data and the predictions derived from the design code standards. Overall, the expressions from ACI 318-11 and Eurocode 2-04 closely approximated the splitting tensile strength of the mixes, whereas other models notably underestimated the experimental results. The data suggest that in the MP0 mix, the ACI 318-11 and Eurocode 2-04 expressions tend to slightly overestimate the experimental results, highlighting the detrimental impact of metakaolin on the splitting tensile strength of the concrete. Conversely, in mixes containing the wastepaper fiber, these expressions slightly underestimate the results, indicating the beneficial influence of the wastepaper fiber on enhancing the splitting tensile strength of the concrete mixes.

### 3.4. Water Absorption

**Figure 9:**
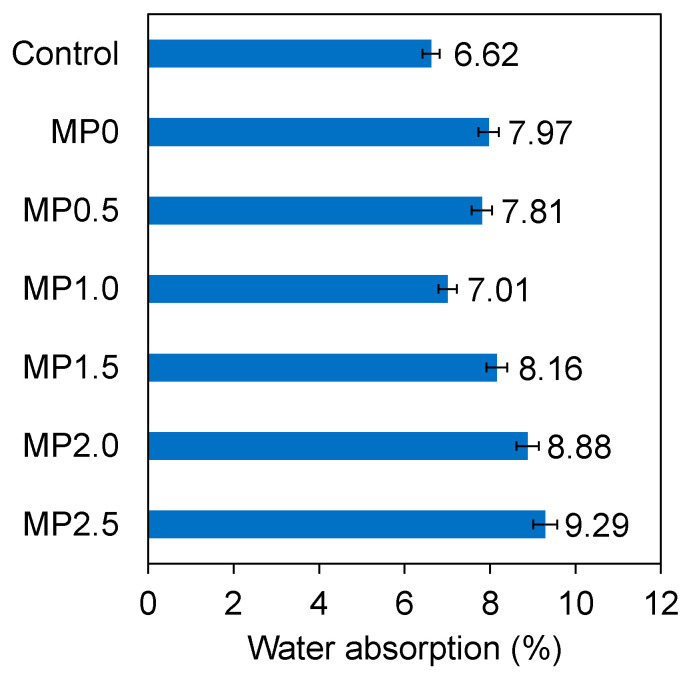
Water absorptions of different mixes.

Figure 9 represents the 28-day water absorption of various mixes. According to the results, inclusion of 20% metakaolin resulted in an increase in the water absorption of the mix by about 20%. This observation is consistent with the previous research finding on metakaolin inclusion in cement-based concrete by up to 20% (by mass of cement), which demonstrated that while the presence of metakaolin beneficially reduces the capillary action in the concrete, it slightly increases the absorption levels when measured by total immersion [57]. This phenomenon can be related to the increased total pore volume of concrete when metakaolin is introduced. As will be shown by the SEM analysis, since metakaolin particles are very small and possess high surface energy, they tend to cluster together. These clusters are essentially random assortments of particles with low density, and they enclose trapped air. Consequently, under these conditions, the water absorption of concrete increases compared to the conventional concrete [52]. Khatib and Clay [57] examined the water absorption properties of metakaolin mixes and determined that substituting 15% of the cement with metakaolin in both paste and concrete resulted in an approximately 10% increase in the pore volume for the paste and a 20% increase in the water absorption for the concrete [57].

According to Figure 9, incorporation of wastepaper fiber by up to 1% into the concrete containing metakaolin resulted in a decrease in the water absorption of the concrete by about 12%. This finding is in agreement with previous studies [71,72]. According to the SEM images which will be presented under microstructural analysis, the incorporation of 1% wastepaper fiber resulted in a significant decrease in the quantity of pores within the concrete structure. Additionally, the introduction of 1% wastepaper fibers led to a decrease in the number of microcracks within the mix in contrast to the mix with no fiber. This observation can be attributed to the even distribution of the fibers and their advantageous bridging effect within the concrete matrix. The decreased water absorption observed in mixes incorporating wastepaper fiber, compared to the unreinforced mix, is attributed to the fibers limiting the formation and propagation of microcracks. This restriction contributes to a denser microstructure in the concrete, reducing permeable voids [44]. Ahmedizat et al. [71] investigated the fabrication of eco-friendly concrete by incorporating recycled, shredded printing wastepaper. Their findings revealed a 5% decrease in the water absorption of the concrete containing 0.6% paper fibers (by volume of concrete) after 28 days compared to the plain concrete. This decrease was attributed to the microstructure modification of concrete which resulted in the decreased porosity of the wastepaper modified mixes. Jamshaid et al. [53] examined the impact of natural cellulosic fibers on water absorption of the concrete. They observed that the incorporation of 2% (by weight of cement) jute and sisal fiber led to a decrease of approximately 13% in the water absorption, owing to the denser and less porous mix by incorporating the fiber, which hindered the movement of the liquid.

The results in Figure 9 also demonstrate the influence of higher wastepaper fiber content on the water absorption of the metakaolin concrete mix. When analyzing the water absorption capacity of the mixes containing 1.5% (MP1.5), 2% (MP2.0), and 2.5% (MP2.5) wastepaper fibers, a trend of increasing water absorption with higher fiber content is evident. For the MP1.5 mix, a 1.5% inclusion of wastepaper fibers resulted in an 8.16% water absorption rate, suggesting a complex interaction between the fibers and the cementitious matrix. Based on the SEM analysis, which will be discussed later, wastepaper fibers may begin to agglomerate, thereby creating localized zones of weakness and increased porosity. This phenomenon could account for the slightly increased water absorption rates, compared to the mix with 1% fiber, which exhibited a denser microstructure. The trend continued with MP2.0 and MP2.5 mixes, which showed water absorption rates of 8.88 and 9.29%, respectively. These values indicate a further increase in the absorption, potentially attributable to the volumetric proportion of fibers creating a more heterogeneous matrix. The hydrophilic nature of the wastepaper fibers may contribute to the increased water absorption. The inherent characteristics of the fibers to absorb water can result in a higher water retention capacity within the concrete, which is particularly notable in the mixes with a 2 and 2.5% fiber content. This effect is likely to be more pronounced in these mixes due to the greater quantity of fibers present.

### 3.5. Drying Shrinkage

**Figure 10:**
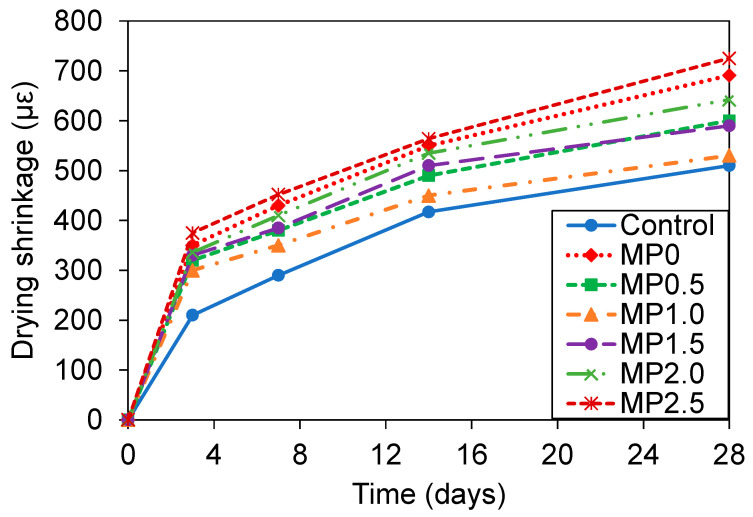
Variation of drying shrinkage of different mixes with time.

Figure 10 shows the drying shrinkage results of different mixes at different curing ages. According to the results, applying 20% metakaolin instead of the cement increased the 28-day drying shrinkage of the concrete by about 26%. This is in agreement with a prior research study on the shrinkage development of concrete modified with supplementary cementitious materials, including metakaolin [73]. The increased drying shrinkage in concrete can be ascribed to the higher porosity resulting from the introduction of metakaolin. This greater porosity is a result of the small size and increased surface energy of the metakaolin particles, which tend to aggregate. These aggregates consist of randomly arranged particles with low density, resulting in the entrapment of air and creation of a permeable void with the matrix [52]. Kou et al. [73] examined the effects of different mineral additives, including metakaolin, on concrete. Their findings demonstrated that substituting 15% of cement with metakaolin led to an approximately 11% increase in the drying shrinkage of concrete after 112 days of curing.

Referring to Figure 10, the incorporation of wastepaper into the metakaolin-infused concrete resulted in a decrease in the drying shrinkage of the concrete. Incorporating 0.5 and 1% wastepaper into the mix resulted in a decrease of approximately 13 and 23% at 28 days, respectively, when compared to the plain metakaolin mix. Notably, the drying shrinkage of the mix with 1% wastepaper closely resembled that of the conventional concrete. This observation is consistent with earlier research findings on shrinkage development of wastepaper fiber-reinforced cement paste [13] and mortar [74]. The decreased drying shrinkage of the mixes with incorporation of the wastepaper fiber of up to 1% compared to the plain metakaolin mix could be due to the fibers restricting the formation and spread of microcracks, thereby decreasing the formation of permeable voids and capillary channels in the mix and resulting in a more compact concrete microstructure [44,75]. Nonetheless, further introduction of wastepaper fiber ranging from 1 to 2.5% resulted in roughly a 37% increase in the drying shrinkage of the concrete at 28 days. As will be discussed in detail by SEM, an overabundance of the wastepaper fibers incorporated into the mix tended to group the fibers together within the concrete matrix. This aggregation of the fibers hindered their effective dispersal and bridging potential, ultimately leading to greater porosity and water flow, which in turn, contributed to the increased drying shrinkage.

Jiang et al. [13] evaluated the shrinkage behavior of wastepaper fiber-reinforced cement paste and found that by adding 0.2% pre-processed newsprint wastepaper fiber the drying shrinkage of the concrete at 28 days decreased by about 48%. They ascribed this observation to the enhancement of the hydration process and the decrease in the cumulative pore volume facilitated by the inclusion of wastepaper fibers within the mix. The enhancement of the hydration process by wastepaper fibers was linked to the intrinsic characteristics of the cellulosic fibers. These fibers are hydrophilic and can absorb water, which is then gradually released into the cement matrix during the early stages of hydration. This internal source of water supports the cement hydration process, especially in conditions where external moisture may not be readily available or uniformly distributed throughout the concrete. The slow release of water from the fibers fosters a more continuous hydration process, allowing for the formation of calcium silicate hydrate over a prolonged period, thereby enhancing the interfacial transition zone between the fibers and the cementitious matrix [13,76]. The porous nature of the wastepaper fibers acts as a micro-reservoir of water within the concrete. During the critical phase of hydration, when water is being consumed rapidly, the fibers release their stored water, reducing the risk of self-desiccation within the capillaries of the concrete. This self-curing mechanism ensures that the hydration process proceeds more effectively, leading to an increase in the compactness of the concrete and a reduction in its cumulative pore volume. Consequently, the concrete matrix becomes denser, improving its permeability resistance, and, ultimately, its durability and service life [77,78]. Arnaud et al. [74] conducted a study focusing on the characterization of cementitious mortar by incorporating wastepaper fibers. Their research revealed a decrease of approximately 76% in the 28-day drying shrinkage of the mortar when the wastepaper was added at a ratio of 1/16 of the weight of sand. In another study regarding the utilization of cellulose fiber in cement paste, it was found that introducing 0.5% highly refined cellulose fiber (known as ultra cellulose fiber with a chemical formula (C_6_ H_12_ O_5_)*_n_*) by the weight of cement resulted in a decrease of approximately 35% in the 28 days drying shrinkage [79]. Zhang et al. [80] analyzed how the addition of coir as a cellulosic fiber affected the drying shrinkage of cement paste and indicated that incorporating 4% fiber by the weight of cement decreased the 28-day drying shrinkage by approximately 50%.

### 3.6. Microstructural Analysis

**Figure 11:**
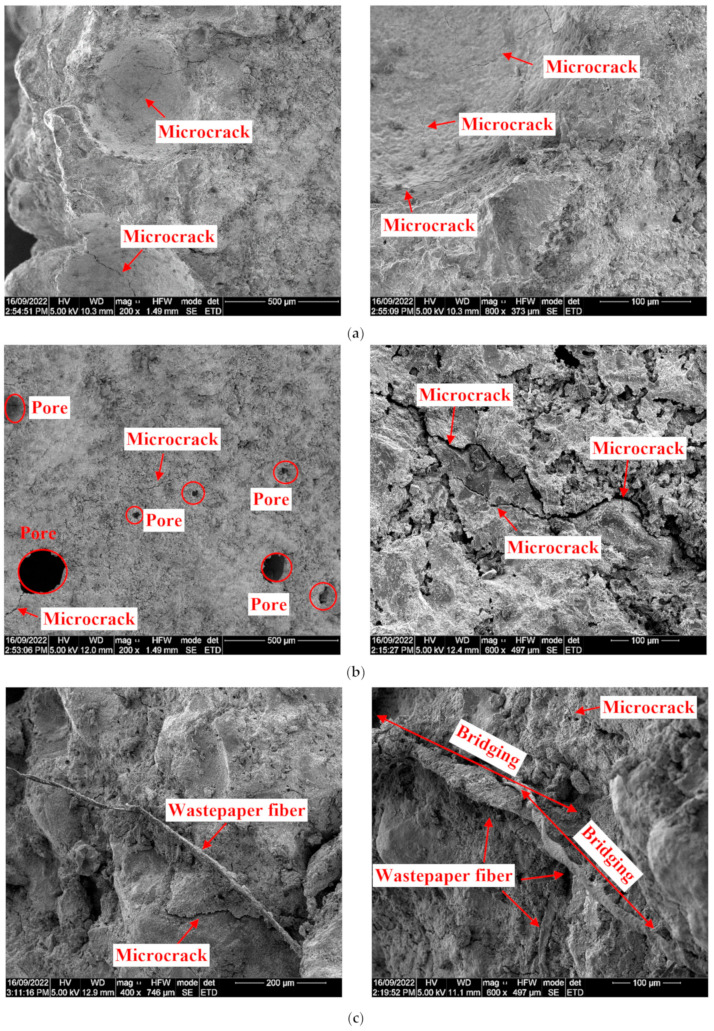

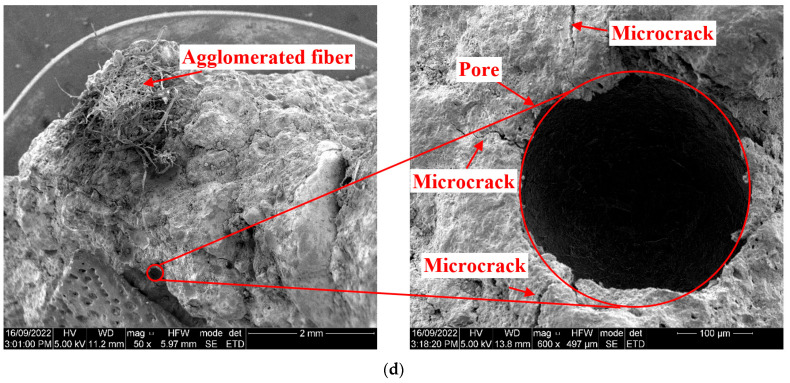
SEM images of (**a**) control, (**b**) MP0, (**c**) MP1, and (**d**) MP2.5 mixes.

Figure 11a–d depicts the SEM images of different concrete mixes containing metakaolin and wastepaper fiber. As evident in Figure 11a,b, substituting cement with metakaolin adversely affected the microstructural cohesiveness of the concrete, referring to the uniformity and connectivity of the hydrated cement matrix and the degree to which it is free of voids, cracks, and unreacted materials. These characteristics are critical to the overall mechanical performance and durability of concrete. The inclusion of 20% metakaolin resulted in an increase in the quantity of pores along with a greater occurrence of microcracks in contrast to the control mix characterized by a denser structure. Comparing these figures reveals that the inclusion of metakaolin, known for its very fine particles and high surface energy that tend to cluster together, resulted in a decrease in the overall densification and bonding within the concrete matrix, leading to an increased number of pores and microcracks. Figure 11c illustrates the influence of incorporating 1% wastepaper fiber into the metakaolin mix. As shown, the inclusion of 1% wastepaper fiber led to a reduction in the number of pores within the concrete. This can be ascribed to the even dispersion of the fibers and their beneficial bridging influence within the concrete matrix. Furthermore, the introduction of 1% wastepaper fiber resulted in a decrease in the quantity of microcracks within the mix in contrast to the mix without wastepaper fiber. These observations provide insight into why concrete mixes including the wastepaper fiber displayed a decreased water absorption, decreased drying shrinkage, and enhanced compressive and splitting tensile strengths when compared to plain metakaolin mix. Conversely, as can be observed in Figure 11d, when 2.5% wastepaper fiber was introduced, there was a notable tendency for the fibers to aggregate and cluster within the concrete matrix. This fiber aggregation created substantial voids and pores in the concrete structure, resulting in an uneven distribution of stress within the matrix. This, in turn, led to a decrease in the compressive and splitting tensile strengths and an increase in the drying shrinkage and water absorption of the metakaolin mixes.

Solahuddin et al. [81] carried out an assessment of the structural performance of reinforced concrete beams that incorporated shredded wastepaper as an additive. Their SEM images indicated that the inclusion of up to 10% shredded wastepaper led to a reduction in the pores and voids within the concrete matrix. Xu et al. [82] investigated the impact of synthetic cellulose fibers on mechanical characteristics of cement concrete. They demonstrated that the presence of the cellulosic fibers, uniformly distributed without any clumping, enhanced the bonding between the cementitious materials in the concrete. In the SEM examination carried out by dos Santos et al. [83] to explore the influence of sugarcane bagasse fibers in mortar, a consistent dispersion of the fibers within the mortar matrix was noted. This uniform distribution resulted in a robust connection between the fibers and the cementitious substance. Based on the SEM micrographs captured by Bentchikou et al. [84], an increased content of recycled packaging box wastepaper fiber caused uneven dispersion, fiber agglomeration, and congestion. Consequently, this led to a higher presence of air voids within the cement composite, ultimately adversely affecting the mechanical properties of the composite.

**Figure 12:**
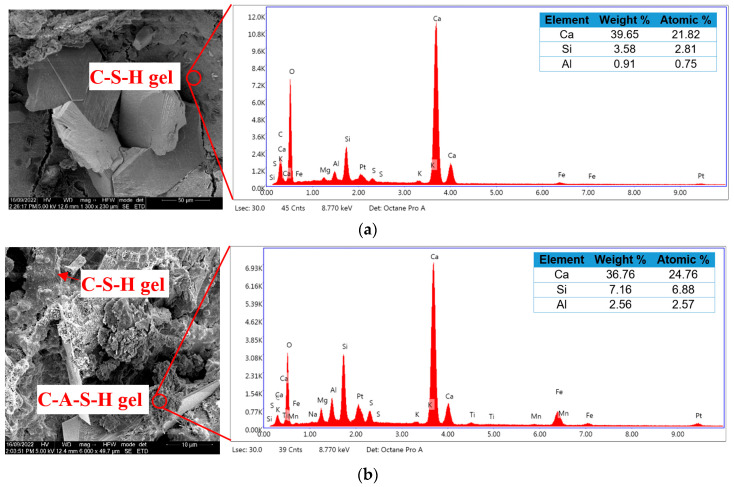
EDS analysis of (**a**) control and (**b**) MP0 mixes.

In Figure 12, the findings from the EDS analysis of various concrete mixes are presented. By comparing the graph and elemental composition shown in Figure 12a,b it is clear that the average Si and Al intensities in the MP0 mix was higher than those in the control mix. The Ca:Si atomic ratio in the control mix was measured as 7.76, whereas in the MP0 mix the Ca:Si atomic ratio decreased to 3.59. This ratio decrease can be ascribed to the replacement of cement with metakaolin in the MP0 mix, which increased levels of Si and Al compared to the cement. Additionally, the figures indicate the presence of calcium silicate huydrate (C-S-H) gel in the control mix, while the EDS results reveal the formation of calcium aluminum silicate hydrate (C-A-S-H) gel in the MP0 mix as well due to the higher proportions of Si and Al in the metakaolin mix and the pozzolanic reaction. Kavitha et al. [85] conducted an EDS analysis for concrete with varying percentages of metakaolin and found that the Ca:Si ratio decreased from 2.14 in the control mix to 1.84 in the mix with a 10% replacement of cement with metakaolin. Zhao and Khoshnazar [14] assessed the microstructure of cement paste incorporating metakaolin and suggested that the introduction of metakaolin caused a transformation from a predominantly CaO-based composition to more of a composition of CaO-SiO_2_-Al_2_O_3_.

## 4. Conclusions

In this research, mechanical, durability, and microstructural characteristics of concrete incorporating metakaolin as a replacement for cement and plasterboard wastepaper fiber as an additive have been investigated. The following conclusions can be drawn:

- Incorporating wastepaper fiber into metakaolin-based concrete leads to a decrease in the workability of the concrete, with a 67% decrease observed at a 2.5% fiber content. This workability decrease can be attributed to the ability of the fiber to absorb water and its rough texture.
- Replacing 20% of cement with metakaolin results in a 24% decrease in the compressive strength, a 26% decrease in the splitting tensile strength, a 20% increase in the water absorption, and a 35% increase in the drying shrinkage of the concrete at 28 days. These changes are primarily caused by the increased porosity and the promotion of crack initiation and propagation within the metakaolin-based concrete.
- The addition of wastepaper fiber to metakaolin concrete up to an optimal content of 1% fiber causes a 29% increase in the compressive strength, a 38% increase in the splitting tensile strength, a 12% decrease in the water absorption, and a 23% decrease in the drying shrinkage of the concrete at 28 days. According to microstructural analyses, these improvements in the mechanical and durability properties of the metakaolin concrete are attributed to the uniform dispersion of the fibers within the concrete matrix when they are used at up to an optimal level, thereby effectively serving as bridges to obstruct the propagation of microcracks.
- Increasing the wastepaper fiber content from 1 to 2.5% results in a 33% decrease in the compressive strength and a 21% decrease in the splitting tensile strength, along with a 32% increase in the water absorption and a 37% increase in the drying shrinkage of the metakaolin concrete at 28 days. This can be explained by the clumping of the fibers and their uneven distribution within the concrete matrix as observed in the microstructural analyses.

Results show that the concrete containing 20% metakaolin as a binder and 1% wastepaper fiber exhibited comparable mechanical and durability properties to conventional concrete. This is promising for reducing the reliance on conventional cement by incorporating waste materials like waste plasterboard, while also utilizing by-products such as metakaolin. Such practices in the building industry promote resource conservation, reduce carbon emissions, and help mitigate the environmental impact associated with conventional concrete production. Moreover, the utilization of wastepaper helps diminish the quantity of waste plasterboard typically disposed of in landfills. 

Future research is recommended to assess the pozzolanic activity of metakaolin when used as a replacement of cement in wastepaper fiber-reinforced concrete. When employing natural fibers such as wastepaper, it is recommended to conduct a degradation analysis, including the effects of wetting and drying cycles on wastepaper concrete. Additionally, in examining the mechanical behavior of fiber-reinforced concrete, particularly in tensile testing, it is crucial to understand not only the strength of the concrete but also factors such as stress, specific deformation behavior, and energy absorption. This information is directly relevant to the utilization of low modulus fibers like wastepaper.

## Figures and Tables

**Table 1:** Chemical composition of binders in %.

Composition	Cement	Metakaolin
SiO_2_	19.9	59.0
Al_2_O_3_	4.79	25.4
Fe_2_O_3_	3.14	1.10
CaO	63.3	0.10
MgO	2.03	1.00
Na_2_O	0.29	0.03
K_2_O	0.40	3.00
SO_3_	2.69	–
P_2_O_5_	0.04	–
LOI	3.42	–

**Table 2:** Concrete mix proportions.

Mix ID	Wastepaper Fiber (% wt)	Binder		Sand (kg/m^3^)	Coarse Aggregate (kg/m^3^)	Superplasticizer (% wt)	w/b
		Cement (kg/m^3^)	Metakaolin (kg/m^3^)				
Control	0	330	0	1030	525	0.75	0.6
MP0	0	264	79	1030	525	0.75	0.6
MP0.5	0.5	264	79	1030	525	0.75	0.6
MP1.0	1.0	264	79	1030	525	0.75	0.6
MP1.5	1.5	264	79	1030	525	0.75	0.6
MP2.0	2.0	264	79	1030	525	0.75	0.6
MP2.5	2.5	264	79	1030	525	0.75	0.6

**Table 3:** Design expressions of conventional concrete recommended by existing codes for splitting tensile strength.

Code	Splitting Tensile Strength (MPa)
ACI 318-11 [65]	$0.53\sqrt{{f’}_{c}}$
Eurocode 2-04 [66]	$0.3{\left({f’}_{c}\right)}^{\frac{2}{3}}$
JSCE-07 [67]	$0.44\sqrt{{f’}_{c}}$
NZS 3101 [68]	$0.44\sqrt{{f’}_{c}}$
JCI-08 [69]	$0.13{\left({f’}_{c}\right)}^{0.85}$
AS 3600-18 [70]	$0.36\sqrt{{f’}_{c}}$

## Data Availability

Data are contained within the article.
